# A Confusing Coincidence: Neonatal Hypoglycemic Seizures and Hyperekplexia

**DOI:** 10.1155/2014/595412

**Published:** 2014-03-24

**Authors:** Nihat Demir, Murat Doğan, Sanem Yılmaz, Erdal Peker, Keziban Bulan, Oğuz Tuncer

**Affiliations:** ^1^Department of Pediatrics, Division of Neonatology, Yuzuncu Yil University School of Medicine, 65200 Van, Turkey; ^2^Hospital of the Yuzuncu Yil University, 65250 Van, Turkey; ^3^Department of Pediatrics, Division of Endocrinology, Yuzuncu Yil University School of Medicine, 65200 Van, Turkey; ^4^Department of Pediatrics, Division of Neurology, Ege University School of Medicine, İzmir, Turkey

## Abstract

Hyperekplexia is a rare, nonepileptic, genetic, or sporadic neurologic disorder characterized by startle responses to acoustic, optic, or tactile stimuli. Genetic defects in glycine receptors as well as encephalitis, tumors, inflammation, and disgenesis are among the etiologic causes of the disease. The main problem in hyperekplexia is the incomplete development of inhibitory mechanisms or exaggerated stimulation of excitatory mediators. Hyperekplexia is often confused with epileptic seizures. Here we present a case with hypoglycemic convulsions coexisting with hyperekplexia, causing diagnostic difficulty.

## 1. Introduction

Hyperekplexia (HE), first described by Kirstein and Silfverskiold in 1958, is a rare and nonepileptic clinical entity characterized by exaggerated and generalized startle responses to acoustic, optic, or tactile stimuli [1]. Minimal stimuli can cause severe jerk-like movements in all the limbs. The symptoms of the disease can lead to unavoidable falls with no loss of consciousness that often diminish with age or continue until adulthood.

Glycine is one of the major inhibitory neurotransmitters in the central nervous system. The glycine receptors cause postsynaptic hyperpolarization and synaptic inhibition through chlorine channels in brain and brain stem. The main pathologic impairment is the inability of glycine, which is one of the major inhibitory neurotransmitters in the central nervous system, to display its inhibitory effects, particularly in the brain stem [2]. Hyperekplexia is known to be genetic or sporadic with the genetic form being more frequent. Genetic studies have shown that most of the genetic cases are autosomal dominant and that the responsible gene is found in the alpha-1 and beta subunits and glycine-carrying parts of glycine receptors as well as in the proteins of gephyrin and collybistin that both have glycine-like effects [3]. The sporadic cases of HE are rare and are either idiopathic or due to factors such as encephalitis, tumors, inflammation, and disgenesis [4].

Hypoglycemia is a frequent problem in the newborn. Serious hypoglycemia can lead to optic and mental disorders, epilepsy, and brain damage. Hypoglycemia causes convulsions by increasing glutamate, the main excitatory neurotransmitter of the brain [5].

## 2. Case

The baby boy was born full-term by spontaneous vaginal delivery to a 28-year-old healthy female mother as a fifth pregnancy. Immediately after birth, the infant cried but displayed no change in skin color, and his Apgar scores were 7 and 9 at one and 5 minutes, respectively. There was no history of a consanguineous marriage between the parents or the presence of a similar disease in the other children of the family or other family members. At birth, his weight was 2850 grams (10–50 percentile), height was 50 cm (50 percentile), and his head circumference was 34 cm (50 percentile). In his physical examination, there was no dysmorphic appearance and the other systems were found to be normal. On the postnatal second day, tonic and abnormal jerk-like seizures involving all the extremities were observed. The blood glucose was measured at 32 mg/dL during these seizures. The patient was diagnosed with hypoglycemic convulsion and given phenobarbital, after which he was sent to our clinic and hospitalized. His blood glucose was 32 mg/dL. The patient initially had subtle seizures like pedaling, licking lips, and swallowing which were then followed by generalized tonic convulsions. The patient first received 4 cc/kg intravenous 10% dextrose solution. Upon prolonged apnea and deterioration of his general condition both enteral and parenteral nutrition were started. Hormone tests, carried out to explain his resistant hypoglycemia, yielded normal results. Respiratory ventilation with continuous positive airway pressure (CPAP) was required for the pathologic apnea following convulsions. In the meantime, his cranial magnetic resonance imaging (MRI) and electroencephalogram (EEG) results were normal. Due to the persistence of the convulsions, midazolam infusion was given. In spite of the maximum dose of midazolam infusion and vitamin B6 supplements, the convulsions continued and levetiracetam was added to the treatment regimen. The urine and blood amino acids, the urine sulphite test, and the serum uric acid were all within normal ranges. Convulsions continued despite the normalization of the blood sugar level and the multiple antiepileptic drugs. Then, an important observation was made. It was observed that the patient was extremely alert to acoustic and tactile stimuli and that these stimuli triggered the convulsions. In the repeated EEG, there was no electroencephalographic abnormality accompanying the seizures triggered by stimuli and no other findings except for movement artefacts. Upon observing that the nasal pillow mask triggered seizures, the diagnosis of hyperekplexia was considered and treatment with clonazepam was started. The midazolam, phenobarbital, and levetiracetam that he had been taking were gradually reduced in dose and stopped. Considering the effects that minimal stimuli had, CPAP was replaced by an oxygen hood for ventilation and stimulating interventions were minimalized. The patient then displayed a significant decrease in the intensity and number of his seizures, started normal breast feeding, had normal blood glucose levels, and had no apnea. Where upon, he was discharged from the hospital with clonazepam treatment. During the 4-month follow-up period, he did not display apnea on the apnea monitor and he did not have any convulsions, only minimum-level contractions.

## 3. Discussion

The most important clinical feature of hyperekplexia is extreme reaction to stimuli caused by genetic mutations or incomplete development of the inhibitory glycine receptors in the brain secondary to encephalitis, tumors, inflammation, and dysgenesis [4, 6, 7]. The clinical presentation of hyperekplexia differs with the age of the patient, and this situation leads to difficulty in diagnosis. Particularly in the newborn, the diagnosis is quite difficult because of the many manipulations in the intensive care unit, increased susceptibility to stimuli, and metabolic problems (hypoglycemia, hypocalcemia). Feeding difficulties, life-threatening pathologic apnea, sudden infant death, complete bundle branch block, and cerebral anoxia can occur in serious hyperekplexia [8].

In the differential diagnosis of hyperekplexia; paroxysmal extreme pain disorder, epilepsy, nonketotic hyperglycemia, Crisponi syndrome, and neonatal tetanus should be considered. It has been reported that in seriously affected infants, severe jerk-like spasms can be confused with epileptic attacks and even with status epilepticus. Many studies have interpreted the absence of epileptic activity on EEG during repeating jerks as being indicative of HE [8, 9]. In our case, the diagnosis of hyperekplexia was considered because convulsions continued in spite of corrected hypoglycemia, the EEG findings were normal and jerks diminished significantly with clonazepam treatment. Our hyperekplexia case was differentiated from* neonatal tetanus* by the fact that complaints started immediately after birth and were nonprogressive. The differentiation from* paroxysmal extreme pain disorder* was made because the jerks were triggered with painful stimuli as well as with acoustic and optic stimuli and no change in skin color was observed; and from Crisponi syndrome due to the lack of saliva flow during convulsions, generalized convulsions, and no other additional abnormality; and then nonketotic hyperglycaemia was excluded by the normal amino acid analysis [10].

In hypoglycemia, the main excitatory amino acid glutamate is poorly reabsorbed due to its extreme secretion in the synaptic area and insufficiency of energy-dependent channels and thus leads to increased amounts of secondary extracellular glutamate which in turn induces convulsions [11]. In the neonatal period, the receptors for glutamate (particularly N-methyl-D-aspartate [NMDA] and alpha-amino-3-hydroxy-5-methyl-4-isoxazolpropionic acid [AMPA]) are well developed, while the inhibitory system is still weak [11, 12]. Glycine acts as an inhibitor in the central nervous system and at the same time functions as an excitatory coantagonist [13]. Animal studies have shown that a genetic or experimental defect in glycine receptors increase the susceptibility of NMDA receptors; this high susceptibility and defective inhibitory mechanism leads to extreme stimulation of the excitatory activity and so to uncontrolled seizures and convulsions [14, 15]. Hyperekplexia is primarily caused by a genetic defect in glycine receptors; rarely it can also occur secondary to encephalitis, tumors, inflammation, and dysgenesis. Our patient was a primary case of hyperekplexia, but the initial convulsions secondary to hypoglycemia caused difficulty in diagnosis. The increase in glutamate due to hypoglycemia plus the glycine defect causing hyperekplexia might have significantly increased cortical irritation and thus the hyperplexia, leading to a status epilepticus-like clinical presentation. Further well-designed studies on the relationship between hyperekplexia and hypoglycemia are required.

Although hyperekplexia is a rare nonepileptic phenomenon, it should be considered in cases of convulsions resistant to therapy, in convulsions with no organic underlying disease or in persistent convulsion-like involuntary movements after treatment of the underlying disease. With a correct diagnosis of hyperekplexia, unnecessary antiepileptic therapy and possible side effects can be avoided and appropriate treatment can diminish convulsion attacks.
